# Decompressive craniectomy in aneurysmal subarachnoid hemorrhage: can favorable outcome be achieved?

**DOI:** 10.1007/s00701-025-06485-9

**Published:** 2025-03-11

**Authors:** Teodor Svedung Wettervik, Alba Corell, Merete Sunila, Per Enblad, Fartein Velle, Peter Lindvall, Lars Kihlström Burenstam Linder, Bjartur Sæmundsson, Alexander Fletcher-Sandersjöö, Klas Holmgren

**Affiliations:** 1 Department of Medical Sciences, Section of Neurosurgery, Uppsala University, Uppsala, Sweden; 2 Department of Clinical Neuroscience, Institute of Neuroscience and Physiology at the Sahlgrenska Academy, University of Gothenburg, Gothenburg, Sweden; 3 Department of Neurosurgery, Sahlgrenska University Hospital, Gothenburg, Sweden; 4 Department of Clinical Science - Neurosciences, Umeå University, Umeå, Sweden; 5 Department of Neurosurgery, Karolinska University Hospital, Stockholm, Sweden; 6 Department of Clinical Neuroscience, Karolinska Institutet, Stockholm, Sweden

**Keywords:** Aneurysmal subarachnoid hemorrhage, Decompressive craniectomy, Intracranial pressure, Outcome, Thiopental

## Abstract

**Background:**

Decompressive craniectomy (DC) is a last-tier treatment for managing refractory intracranial hypertension in patients with aneurysmal subarachnoid hemorrhage (aSAH), though concerns persist about whether it primarily prolongs survival in a state of severe disability. This study investigated patient characteristics, surgical indications, complications, and outcomes following DC in aSAH.

**Methods:**

In this Swedish, retrospective multi-center study, 123 aSAH patients treated with DC between 2008–2022 were included. Data collection included demographic details, aSAH characteristics, injury severity, DC indication, complications, and outcome at roughly six months post-DC (modified Rankin scale [mRS]) dichotomized as survival vs. mortality (0–5 vs. 6) and favorable vs. unfavorable (0–3 vs. 4–6).

**Results:**

The median age was 53 years and 66% were females. Two thirds presented with a WFNS grade 4–5 and 83% with a Fisher grade 4 hemorrhage. Most aneurysms were located at the middle cerebral artery (65%) and treated with clip ligation (59%). DC significantly reduced midline shift from 9 to 2 mm and obliteration rates of basal cisterns from 95 to 22% (*p* < 0.05). Reoperation for hematomas or extension of the DC were rare (< 5%). At follow-up, 20% were deceased, while 33% had recovered favorably. In univariate logistic regressions, younger age was associated with favorable outcome and reduced mortality. Other patient demographics, injury severity, and factors related to the DC surgery lacked association with outcome.

**Conclusions:**

aSAH patients treated with DC presented with severe primary brain injuries and signs of intracranial hypertension. DC resulted in radiological improvements regarding mass effect and a low rate of postoperative complications. Although the results were based on a selected population of aSAH patients, an encouraging rate of favorable outcome was found, particularly among younger patients. However, the absence of additional outcome predictors underscores the ongoing challenges in improving patient selection for DC in aSAH.

## Introduction

Aneurysmal subarachnoid hemorrhage (aSAH) continues to carry a high mortality and morbidity rate [30]. Modern management focuses on early aneurysm occlusion to prevent rebleeding, cerebrospinal fluid (CSF) drainage to treat acute hydrocephalus, and vigilant efforts to prevent delayed cerebral ischemia (DCI) [9, 11]. Furthermore, neurocritical care (NCC) aims to optimize cerebral physiology, particularly by controlling intracranial pressure (ICP) to prevent brain herniation and ischemia [8, 9, 35, 38, 39]. Elevated ICP is common after aSAH, occurring both immediately after ictus and later during the acute phase due to rebleeding, hydrocephalus, and brain edema [24, 31, 36, 47]. Intracranial hypertension may be indicated by a deterioration in consciousness noted in neurological examinations and presence of mass effect on imaging. In addition, unconscious aSAH patients who are intubated are typically monitored for ICP using either an external ventricular drainage (EVD) or an intraparenchymal probe when repeated neurological examinations are less feasible. In these situations, maintaining ICP below 20 mmHg is generally targeted, although robust evidence supporting specific ICP thresholds in aSAH remain scarce [31, 34, 35, 38]. NCC strategies focus on both preventing and actively managing intracranial hypertension through interventions such as CSF drainage, hematoma evacuation, controlling pCO₂, optimized sedation, and hyperosmolar therapy [8, 31, 38]. Despite these first-line measures, approximately 10% of aSAH patients develop refractory intracranial hypertension, for whom last-tier interventions, including barbiturates and decompressive craniectomy (DC), may be considered [3, 4, 6, 10, 12, 15, 19, 25, 44]. The role of DC has been extensively studied in randomized controlled trials and meta-analyses for other acute brain injuries [21, 22, 29]. Specifically, DC has become a widespread treatment to alleviate intracranial hypertension in traumatic brain injury (TBI) [16, 22] and to prevent brain herniation in malignant middle cerebral artery (MCA) infarction, both lowering mortality and resulting in an increased chance of a favorable outcome [21, 29, 42]. In contrast, the role of DC in aSAH remains less well-established, having been primarily evaluated in a limited number of cohort studies [3, 10, 12, 15, 18, 44] that have demonstrated rather poor long-term results with only 10–15% reaching favorable outcome in two recent studies [6, 44]. Notably, aSAH patients treated with DC are in general older with more comorbidities than TBI patients, and often sustain both a global injury at ictus and secondary focal injuries from aneurysm treatments and DCI while DC-treated patients with MCA infarctions exhibit more focally restricted brain injuries. These factors may predispose to a reduced baseline capacity for functional recovery and result in a more severe and widespread cerebral insult compared to other acute brain injury subtypes. Consequently, significant concerns persist regarding whether DC can result in meaningful recovery in aSAH patients with refractory intracranial hypertension or merely prolong survival in a state of severe disability.

To address these questions, the primary aim of this Swedish multi-center study was to investigate the indications for DC and its ability to reduce mass effect and lead to favorable outcomes following aSAH. Based on these data, a secondary aim was to identify predictors of outcomes, including mortality. We hypothesized that DC was relatively rare in aSAH management and that the majority had an unfavorable outcome. It was further hypothesized that younger age, less severe primary brain injury, and the absence of DCI could be associated with more favorable outcomes.

## Materials and methods

### Patients and study design

This retrospective observational, multi-center study included all patients who underwent DC following aSAH during a 15-year period (2008–2022) at any of the neurosurgical departments in Gothenburg (2008–2018 only), Stockholm, Uppsala, and Umeå. These centers collectively provide neurosurgical care to a catchment area encompassing 7.5 million people, representing 72% of the Swedish population.

### Treatment protocol

Patients with aSAH were admitted to the neurosurgical department within their respective catchment areas. All centers generally adhered to treatment principles outlined in international guidelines [17, 39], though variations between local management protocols existed [2]. Aneurysms were typically treated as early as possible, either by endovascular intervention or surgical clipping. Significant intracerebral hemorrhages (ICH) were surgically evacuated, and all patients started administration of nimodipine upon admission. Poor-grade aSAH patients (World Federation of Neurosurgical Societies [WFNS] grade 4–5) were typically intubated, mechanically ventilated, and sedated. Unconscious patients and awake patients with hydrocephalus underwent placement of an external ventricular drainage (EVD). DCI was defined as focal neurological deficits or a decline in consciousness occurring at a delayed time point from ictus, not attributable to complications such as rebleeding, hydrocephalus, or meningitis. In cases of suspect DCI, modified HHH (hypertension, hypervolemia, and hemodilution)-therapy, as well as endovascular interventions (intra-arterial vasodilation or balloon angioplasty), were considered [13]. The modified HHH approach primarily focused on induced hypertension, near-zero fluid balance (with strict avoidance of hypovolemia), and generous administration of colloid fluids, though with some variation among centers. At some institutions, vasospasm treatment was avoided at the manifestation of cerebral infarctions and/or difficulties to control ICP. Furthermore, ICP was typically maintained below 20 mmHg, and cerebral perfusion pressure (CPP) above 60–70 mmHg. Basal ICP-lowering treatments included head of bed elevation to 30° at most centers, avoiding hypercapnia, CSF drainage via an EVD, and sedation (primarily propofol or midazolam, with barbiturates employed as a second-tier treatment for refractory ICP).

Primary DC without ICP monitoring was performed early on in cases of severe mass effect or perioperative brain swelling, typically in conjunction with surgical clipping and evacuation of an ICH. Secondary DC was reserved as a last-tier intervention for refractory intracranial hypertension. Hemicraniectomy was the preferred approach for lateralized brain injury or swelling with midline shift, whereas bifrontal DC was employed for diffuse bilateral brain swelling without midline shift. The main objective in all DCs was maximal decompression, achieved through the removal of a large bone flap with duraplasty.

### Data acquisition

Clinical, radiological, and outcome data were collected from medical records and radiological imaging. Demographic variables included age and sex. The severity of the primary injury was assessed using clinical (WFNS) and radiological (Fisher) grades. Pupillary reactivity at admission was classified as normal, one unreactive pupil, or two unreactive pupils. Data on aneurysm location and treatment modality were recorded, along with information on surgical evacuation of ICH, ICP monitoring, and barbiturate treatment.

The extent of mass effect, quantified by midline shift (mm) and the status of the basal cisterns (categorized as open, compressed, or obliterated), was assessed using head computed tomography (CT) scans obtained before and after DC. The type of DC (hemicraniectomy vs. bifrontal craniectomy) and the area of decompression (cm^2^) were also evaluated. The latter was determined by measuring the anteroposterior width (from tabula externa) and craniocaudal height of the decompression on CT imaging [32, 37]. Postoperative complications were systematically analyzed, including reoperations for postoperative hematomas, extensions of the cranial decompression, surgical site infections, and subdural hygromas. The presence of postoperative external brain herniation exceeding 1.5 cm [46] and CSF circulation disturbances requiring shunt placement were also recorded.

Lastly, the functional status was assessed by retrospective chart review and determined according to the modified Rankin Scale (mRS) [5] at a time point as close to six months post-DC as possible. Outcome was dichotomized into survival vs. mortality (mRS 0 to 5 vs. 6) and favorable vs. unfavorable (mRS 0 to 3 vs. 4 to 6). All collection of data, including mRS outcomes, was performed by neurosurgical trainees (1–3 per center) with previous experience of chart review and assessment of functional outcomes in similar retrospective study settings. All mRS assessments were based on thorough scrutiny of medical records, including notes on out-patient follow-up visits and telephone follow-ups, while also medical notes from additional in-patient care in close proximity to the six months post-DC endpoint. All information available, including nursing documentation, was used to determine the patient’s ability to live independently (or with community care), ambulate, including their working capacity, and in turn transformed into a functional outcome according to the mRS. In any case where there was insufficient information to determine functional status, the outcome was reported as missing. Also, to ensure consistency among centers, all personnel involved in the collection of data adhered to a pre-defined study protocol that included a list of variables and information on how these should be interpreted and reported (including mRS ascertainments) to make the data assessments as uniform as possible.

### Statistical analysis

Statistical analyses were performed using RStudio software (version 2022.12.0). Continuous and ordinal variables were summarized as medians with interquartile ranges (IQR), while categorical variables were reported as counts and proportions. Changes in radiological mass effect, including midline shift and status of the basal cistern, before and after DC were evaluated using the Wilcoxon test or McNemar’s test, depending on the data type. With mortality and favorable outcome as dependent variables, demographic factors, injury severity, and surgical aspects of DC were analyzed using univariate logistic regression, described with odds ratios (OR) and 95% confidence intervals (CIs). A p-value of less than 0.05 was considered statistically significant.

## Results

### Patient characteristics

In the cohort of 123 aSAH-patients treated with DC, the median age was 53 (IQR 45–59) years and 81 (66%) were females (Table 1). At admission, 67% presented with a WFNS grade 4 to 5, while 13% patients exhibited one or two unreactive pupils (missing data, *n* = 1). All patients had a grade 3 or 4 Fisher score and most aneurysms were located at the MCA (65%), followed by the internal carotid artery (ICA; 22%), anterior cerebral artery (ACA; 12%), and the posterior circulation (1%). Most aneurysms were treated with clip ligation (59%), 35% underwent endovascular embolization, 5% received both treatments, and aneurysm occlusion was not feasible in 1%. A majority (54%) of patients had an ICH that required evacuation. Nearly all patients (99%) underwent ICP monitoring, using either an EVD in 63%, an intraparenchymal monitor in 8%, or both in 28% of cases. DCI occurred in 20%, and 46% were treated with barbiturates.

**Table 1:** Demographics, admission variables, clinical course, and functional outcome

Variables	
Patients, *n* (%)	123 (100%)
Age (years), median (IQR)	53 (45–59)
Sex (male/female), *n* (%)	42/81 (34/66%)
WFNS	
Grade 1 to 3, *n* (%)	41 (33%)
Grade 4 to 5, *n* (%)	82 (67%)
Pupillary status (normal/1 unreactive/2 unreactive), *n* (%)	106/11/5 (87/9/4%)
Fisher	
1–2, *n* (%)	0 (%)
3, *n* (%)	20 (16%)
4, *n* (%)	103 (84%)
Aneurysm location	
ICA, *n* (%)	27 (22%)
ACA, *n* (%)	15 (12%)
MCA, *n* (%)	80 (65%)
Posterior circulation, *n* (%)	1 (1%)
Aneurysm treatment	
No treatment, *n* (%)	1 (1%)
Embolization, *n* (%)	43 (35%)
Clipping, *n* (%)	73 (59%)
Both, *n* (%)	6 (5%)
Hematoma evacuation, *n* (%)	67 (54%)
ICP-monitoring	
No ICP-monitoring, *n* (%)	1 (1%)
EVD, n (%)	77 (63%)
Intraparenchymal, *n* (%)	10 (8%)
Both, *n* (%)	35 (28%)
DCI, *n* (%)	23 (19%)
Barbiturates (yes), *n* (%)	56 (46%)
mRS, median (IQR)	4 (3–5)
Favorable outcome, *n* (%)	37 (33%)
Mortality, *n* (%)	23 (20%)

Missing: Pupillary status (*n* = 1), mRS (*n* = 10)

*ACA* Anterior cerebral artery; *DC* Decompressive craniectomy; *DCI* Delayed cerebral ischemia; *EVD* External ventricular drainage; *ICA* Internal carotid artery; *ICP* Intracranial pressure; *IQR* Interquartile range; *MCA* Middle cerebral artery; *mRS* Modified Rankin scale; *WFNS* World Federation of Neurosurgical Societies

### DC – indication, timing, type, size, and impact on mass effect

A minority of cases (8%) underwent primary DC (Table 2), while the majority were secondary DCs performed due to refractory intracranial hypertension and/or radiological mass effect. The median time from ictus to DC was 3 days (IQR 2–5). Nearly all DCs (98%) were unilateral. The median DC area was 103 (IQR 95–112) cm^2^. The extent of midline shift significantly improved after DC for all patients in the cohort, from a median of 9 mm (IQR 5–12) preoperatively to 2 mm (IQR 0–4) postoperatively (*p* < 0.001) (Table 3). Furthermore, basal cisterns were compressed or obliterated in 95% of cases before DC, decreasing to 22% post-DC (*p* < 0.001).

**Table 2:** DC surgery: timing, indication, and complications

Variables	
Time from ictus to DC (days), median (IQR)	3 (2–5)
Indication for DC (primary/secondary), *n* (%)	10/113 (8/92%)
Type of DC (hemi/bifrontal), *n* (%)	121/2 (98/2%)
DC size (cm^2^), median (IQR)	103 (95–112)
Post-DC hematoma, *n* (%)	3 (2%)
Post-DC extension of bony decompression, *n* (%)	3 (2%)
Post-DC external brain herniation, *n* (%)	109 (89%)
Post-DC surgical site-infection, *n* (%)	1 (1%)
Post-DC subdural hygroma, *n* (%)	1 (1%)
Post-DC VP-shunt, *n* (%)	22 (18%)

No missing data

All post-DC hematomas were ipsilateral and extracerebral

*DC* = Decompressive craniectomy; *IQR* Interquartile range; *VP* Ventriculoperitoneal

**Table 3:** Mass effect before and after decompressive craniectomy

Radiological variables	Before	After	*p*
Midline shift (mm), median (IQR)	9 (5–12)	2 (0–4)	***< 0.001***
Basal cisterns (open/compressed/obliterated), *n* (%)	5/67/51 (4/54/41%)	78/35/10 (63/28/8%)	***< 0.001***

No missing data

Bold and italics indicate statistical significance

*DC* = Decompressive craniectomy; *IQR* Interquartile range

### DC – complication rate

A minority of DC cases required reoperation, including evacuation of postoperative hematomas (2%), enlargement of the cranial decompression (2%), evacuation of a subdural hygroma (1%), or revision due to a surgical site infection (1%). External brain herniation (> 1.5 cm) was observed in 89% of the patients, whereas 18% required a shunt at any time point after ictus due to a non-resolved CSF circulation disorder (24% when considering only non-deceased patients).

### DC – functional outcome and prognostic factors

**Fig. 1:**
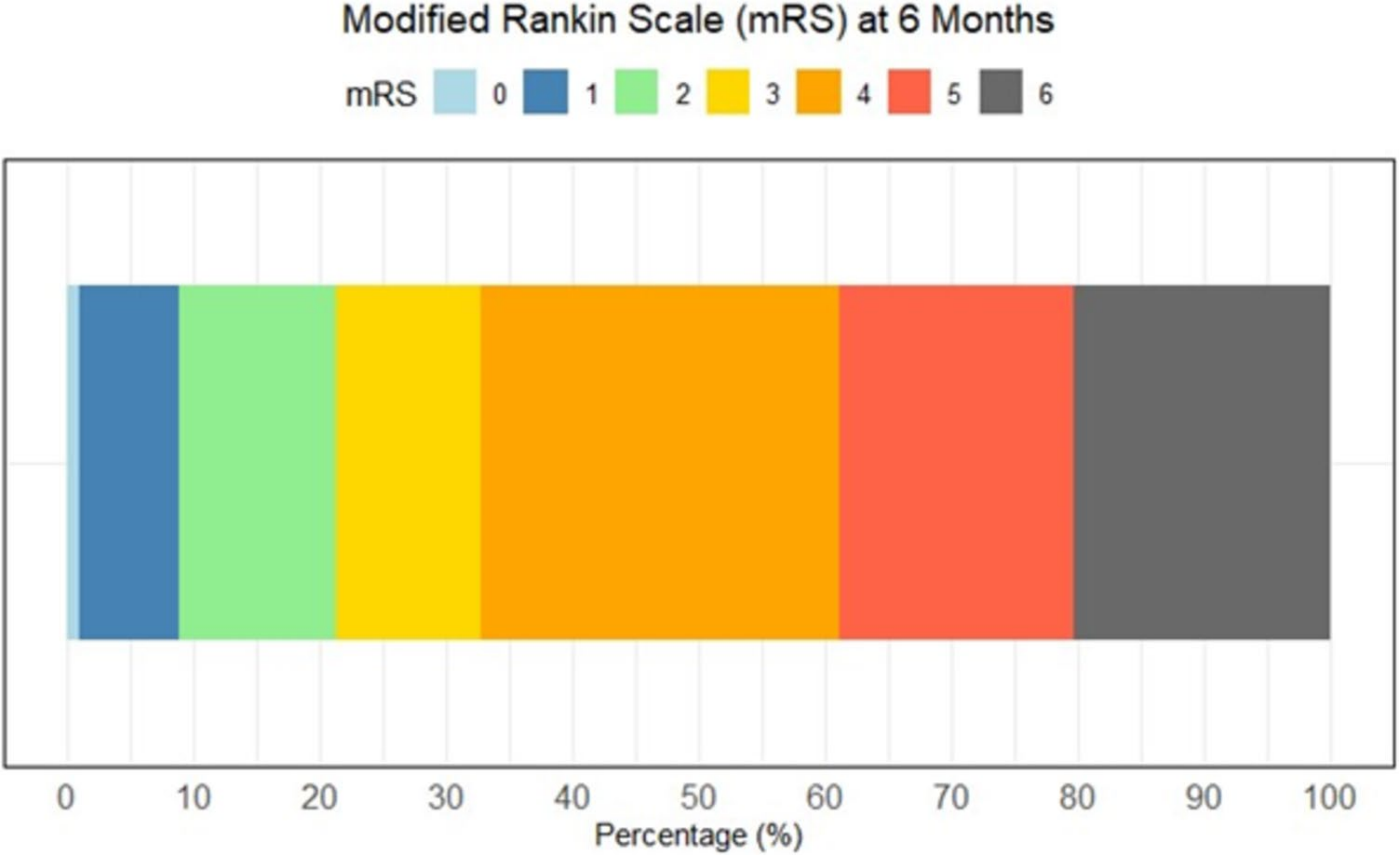
Six-months functional outcome after DC in aSAH. Illustrating the percentage of patients within each mRS category in the aSAH patients treated with DC. Ten patients had no available outcome data and they were excluded from this figure. *aSAH* = Aneurysmal subarachnoid hemorrhage. *DC* = Decompressive craniectomy. *mRS* = Modified Rankin Scale

**Fig. 2:**
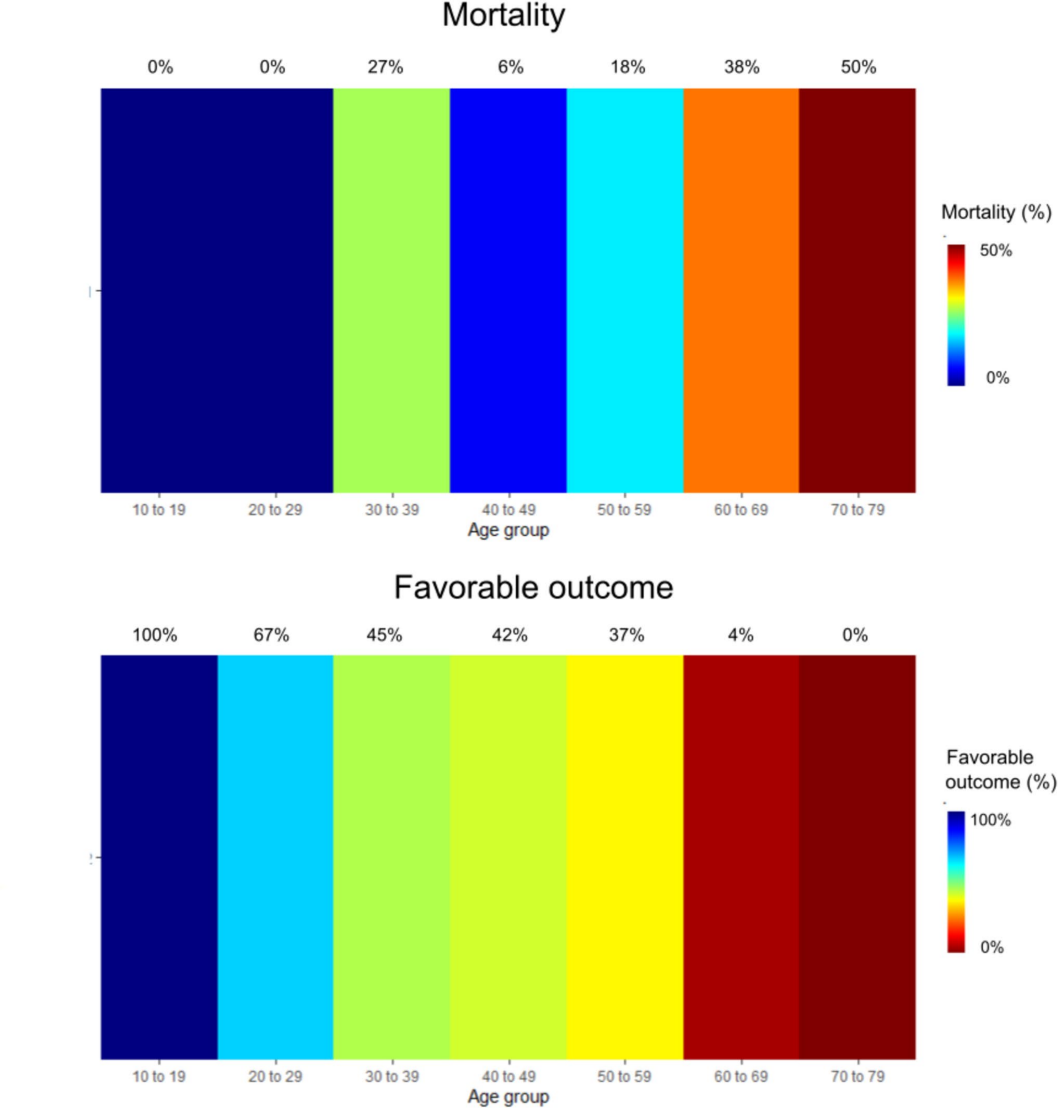
Mortality- and favorable outcome rates for different age categories. Depicting the percentage of patients who were deceased or had reached favorable outcome at follow-up six months after DC with age stratified into decades. Young adolescents and patients in early to middle adulthood (up to 60 years) demonstrated relatively low mortality and high rates of favorable outcomes, whereas individuals above 60 years of age exhibited less favorable recovery. *DC* = Decompressive craniectomy

The median time point for outcome assessment was 6 (IQR 5–11) months post-ictus, of which 10 patients had outcome status reported as missing (8%). At follow-up, the median mRS was 4 (IQR 3–5), with one-third (33%) of patients demonstrating a favorable outcome, and 20% being deceased (Table 1 and Fig. 1). In both the primary and secondary DC sub-cohorts, mortality was 20% (*p* = 0.98), with the rate of favorable outcome slightly higher in the group treated with primary DC (50% vs. 31%, *p* = 0.22). As shown in Table 4, older age was associated with higher mortality (OR [95%CI] = 1.05 [1.01–1.11], *p* = 0.04) and a lower rate of favorable outcome (OR [95% CI] = 0.93 [0.90–0.97], *p* < 0.001). However, no significant associations were identified between mortality or functional outcomes and comorbidities, injury severity, DCI, or the timing and indication for DC. As shown in Fig. 2, mortality was relatively low, and the rate of favorable outcomes was 25% or higher in patients aged below 60 years. In contrast, the majority of patients aged over 60 either survived with a poor outcome or were deceased at follow-up.

**Table 4:** Clinical outcome in relation to demography, injury severity, clinical course, and surgical aspects – univariate logistic regression analysis

Variables	Mortality		Favorable outcome	
	OR (95% CI)	*p*-value	OR (95% CI)	*p*-value
Age (years)	1.05 (1.01–1.11)	***0.04***	0.93 (0.90–0.97)	***< 0.001***
WFNS (grade)	0.88 (0.61–1.29)	0.50	1.05 (0.76–1.48)	0.77
Fisher (grade)	0.52 (0.18–1.65)	0.24	0.68 (0.25–1.90)	0.45
DCI (yes)	1.19 (0.36–3.49)	0.76	1.98 (0.75–5.14)	0.16
Barbiturates (yes)	2.44 (0.97–6.45)	0.06	1.17 (0.53–2.58)	0.70
Time from ictus to DC	0.99 (0.85–1.14)	0.94	0.95 (0.82–1.07)	0.41
Indication for DC (secondary)*	1.02 (0.23–7.12)	0.98	0.45 (0.12–1.72)	0.23
Midline shift (mm) before DC	1.03 (0.94–0.51)	0.51	0.97 (0.89–1.06)	0.56
Basal cisterns before DC (open)	1.0	NA	1.0	NA
Compressed	0.27 (0.03–6.19)	0.30	0.55 (0.06–4.88)	0.57
Obliterated	1.60 (0.19–33–81)	0.69	0.35 (0.04–3.20)	0.32
DC size (cm^2^)	0.99 (0.96–1.01)	0.28	1.02 (1.00–1.04)	0.11

*The statistics show the OR (95% CI) for secondary as opposed to primary DC

Bold and italics indicate statistical significance

*CI* Confidence interval; *DC* Decompressive craniectomy; *DCI* Delayed cerebral ischemia; *NA* Not applicable; *OR* Odds ratio; *WFNS* World Federation of Neurosurgical Societies

## Discussion

In this observational multi-center study of 123 aSAH patients, treatment with DC resulted in immediate radiological improvements in mass effect and a low rate of complications requiring reoperation. Survival at six months was 80%, and 33% achieved favorable outcome. While younger age was associated with better outcomes, other patient- and injury-related predictors of recovery provided limited prognostic information. These results suggest that favorable outcome is possible for a significant proportion of aSAH patients with refractory intracranial hypertension who were treated with DC. However, it is important to stress the lack of a control group of patients with refractory intracranial hypertension who were managed conservatively and that the results must be interpreted in the light of this limitation. Furthermore, the lack of robust prognostic factors, aside from age, highlights the challenges in predicting which aSAH patients that may sustain a favorable outcome after DC.

Only 123 aSAH patients underwent DC over a 15-year period (10 years in Gothenburg) within a catchment area of 7.5 million people. The estimated annual incidence of spontaneous SAH in Sweden is approximately 10 per 100,000 individuals and year [26, 33], with aneurysmal etiology accounting for roughly 75–85% of these cases [26, 43]. Based on these estimates, only 1–2% of the aSAH population within our catchment area were treated with DC, a proportion notably lower than the 10% reported in a recent meta-analysis by Darkwah Oppong et al. [10]. These discrepancies may stem from an overestimated aSAH incidence in our calculations, selection bias in earlier studies (e.g., excluding patients with poor prognoses who were not admitted to tertiary centers), and differences in DC indications across centers. Regardless, it is evident that DC is reserved for a small and highly selected subgroup of aSAH patients.

Demographics of our DC patients, including age and sex, were consistent with general aSAH populations [6], with the majority being female at around 50 years of age, and only two patients older than 70 years, likely reflecting a tendency to withhold DC in older patients due to the anticipated poor prognosis for meaningful recovery. As expected, our data demonstrated that the majority of aSAH patients treated with DC presented with a severe primary brain injury, as reflected by high WFNS and Fisher grades. Consistent with previous studies, most DC patients had an MCA aneurysm with an ICH and underwent surgical clipping with hematoma evacuation. Notably, primary DCs in immediate conjunction with ICH evacuation or surgical clipping were rare (8%). Instead, the majority of DC procedures were performed later, after a median of 3 days from ictus (IQR 2–5). This time interval corresponds to the early effects of the primary brain injury, the progression of brain edema, and potential complications following aneurysm treatment. DCI typically happens later and occurred in approximately 20% of the cases, aligning with rates reported in broader aSAH cohorts [6, 8]. However, we cannot exclude that DCI may have contributed to cytotoxic edema and ICP elevation in some of the DC patients studied herein, not least considering that the presence of substantial early brain injury and associated neurological deficits may have masked the effects of DCI and hence led to underdiagnosis of this complication.

Before DC, radiological imaging demonstrated undoubtable signs of mass effect, including midline shifts and compression of the basal cisterns, which significantly improved postoperatively. DC was also associated with a low surgical complication rate, with reoperations due to postoperative hematomas, subdural hygromas, and surgical site infections found in less than 5% of patients. The median size of the DC was approximately 100 cm^2^, i.e., fairly large, while the need for craniectomy extension at a second stage was rare (Table 2). Most patients exhibited external brain herniation at the craniectomy site, potentially leading to cerebral venous congestion due to impingement against the bony edges of the craniectomy. However, the clinical relevance of this observation and exact mechanism remains unclear [46]. Chronic hydrocephalus requiring shunt placement was relatively frequent in this cohort. While this is a common phenomenon in aSAH in general, DC itself is also known to alter CSF dynamics, including reduced ICP pulse waveforms and impaired resorption, which may independently contribute to hydrocephalus [28]. In keeping with this theory, the 18–24% shunt-dependency rate observed in our cohort (with range depending on if the denominator was based on patients alive at follow-up exclusively or not) was slightly higher than rates reported in broader cohorts of aSAH patients, which include both DC-treated and non-DC-treated individuals, typically ranging from 6–18% [1, 23, 27]. Given the relatively low surgical complication rate of DC in this cohort, its use could potentially be considered earlier in selected cases, particularly in relation to high-dose barbiturate therapy that typically sustains several medical complications [6, 7].

Consistent with the short-term improvements in mass effect after DC, six-months outcomes were highly encouraging with a mortality limited to 20% and 33% of patients achieving favorable outcome. These figures are more favorable than most previous studies on DC in aSAH, with mortality rates ranging from 30–96% and favorable outcomes found in only 4–20% of cases [10, 14, 41, 44], although, some single-center studies have showed more promising results [20]. These differences in DC outcomes may reflect variations in surgical indications and timing. For instance, applying a lower threshold for DC would include patients with less severe injuries, who inherently have a better prognosis. On the other hand, extending the use of alternative ICP management strategies may result in performing DC too late, i.e., at a stage when potentially salvageable neurological injuries have already become irreversible. Additionally, a more conservative approach may influence outcomes after DC, as only patients with a better prognosis are likely to undergo the procedure. The role of DC in aSAH management must also be viewed in a broader context. To obtain successful outcomes, these patients require attentive NCC management throughout the acute phase [38] followed by vigorous neurorehabilitation, to both reduce the burden of secondary brain injury, and to promote early recovery. Variations in six-months outcomes could thus also in part be attributed to differences in these aspects between centers.

As mentioned previously, DC has become a last-tier surgical option for intracranial hypertension in a variety of different acute brain injuries. Of particular interest, the rate of favorable outcome seems to be slightly worse in aSAH patients treated with DC compared to in TBI [22, 45] or in malignant MCA infarctions [21, 29, 45]. Several factors may account for these inconsistencies. One explanation could be that aSAH patients tend to be older than those with TBI and therefore harbor lower functional reserves to recover. Brain atrophy that increases with age also implies that a relatively worse underlying brain injury with more additional edema/hemorrhage volume is required to elicit ICP elevations as when compared to younger individuals. Moreover, aSAH is characterized by both severe global and focal brain injury, of which the global injury typically arises at ictus of the initial bleeding – often accompanied by a transient global hypoperfusion. Focal injuries, on the other hand, commonly occur when aneurysms, particularly in the MCA territory, rupture into the parenchyma, causing an ICH and localized tissue destruction. Treatment of the ruptured aneurysm can also lead to further complications, such as infarctions in downstream vascular territories. Additionally, aSAH patients frequently experience vasospasm and DCI that can entail both focal and widespread infarctions. These focal injuries, especially those involving the MCA territory, often affect motor and language functions that can result in significant disabilities such as hemiparesis and aphasia. The edema in aSAH patients is usually cytotoxic and corresponds to irreversible brain damage, in contrast to TBI wherein vasogenic edema with potentially salvageable brain tissue is more common [40]. Malignant MCA infarction patients also exhibit cytotoxic brain edema, but lack the global brain injury characteristic of aSAH. Altogether, the fact that aSAH patients are generally older, exhibit significant widespread primary and secondary brain injuries, and that the underlying edema often represents cytotoxic swelling due to irreversible brain damage, may contribute to significant morbidity specific for aSAH patients, as compared to DC outcomes after other types of acute brain injuries. Again, nonetheless, results of the present study indicate that favorable results can be achieved with DC for aSAH patients with refractory intracranial hypertension.

Consequently, judicious patient selection seems to be of crucial importance when considering DC in aSAH patients, and in turn suggests identifying predictors of long-term outcome remains a key factor to aid these decisions. To aid these decisions, potential determinants of six-months outcomes among aSAH patients treated with DC were therefore analyzed herein. Regrettably, higher age emerged as the sole risk factor linked to higher mortality and a reduced likelihood of favorable outcomes, particularly for patients above 60 years. Again, this observation likely owes to factors mentioned previously: younger patients generally have greater functional reserves, enhanced capacity for neuroplasticity, and are more susceptible to ICP issues with smaller volumes of edema or bleeding. In contrast, variables related to injury severity (e.g., WFNS or Fisher grades), complications (e.g., DCI), or the timing of DC were not significantly associated with functional outcomes or survival. These findings suggest that the underlying cause and timing of ICP elevation may be less critical for predicting outcomes, as all patients in this cohort ultimately sustained severe brain injuries. This aligns with observations from two smaller studies [6, 44]. A plausible interpretation is that predicting recovery in individual patients remains inherently challenging. In our opinion, however, given the relatively favorable outcomes observed in the current study, it seems reasonable to consider DC in aSAH-patients even when prognostication is uncertain, particularly in those aged under 60.

### Methodological considerations

The primary strength of this study lies in its multi-center design and relatively large cohort. The dataset was mostly near-complete, encompassing detailed information on demographics, aSAH characteristics, treatments, clinical course, and outcomes. Considering the rarity of DC in aSAH, the lack of consensus regarding treatment timing and indications, and ethical concerns around withholding potentially life-saving treatments, conducting large randomized controlled trials on this topic in the near future remains unlikely. Thus, this study provides valuable "real-world" data that contribute to the understanding of DC in aSAH management.

However, there are also several study limitations. The main drawback is the retrospective design and data collection based on historical information, in which selection bias and residual confounding are difficult to account for. In addition, the study included patients from four different centers with slightly different management protocols, e.g., in terms of strategies for aneurysm occlusion, escalation of ICP-lowering treatments, and indication for DC. Also, one of the principal findings – namely the encouraging degree of favorable outcome – was based on outcomes determined by means of retrospective chart review. While these assessments may be subject to inter-rater variability and were based on medical notes that were sometimes summary, several academic neurosurgeons with previous experience of chart review participated in the collection of data, for which patients who could not be categorized with regards to outcome were strictly reported as data missing. Furthermore, while imprecise mRS assessments could theoretically have resulted in minor inaccuracies, all analyses were conducted with mRS outcomes dichotomized between favorable (mRS 0–3) vs. unfavorable (mRS 4–6), i.e., essentially a cut-off between those ambulatory vs those not, and therefore helped to minimize the potential impact of outcome grading precision. While the exact time point for assessment of functional outcome also varied slightly within the cohort, it typically occurred at or later than six months post-DC, and thus allowed for recovery to take place, while otherwise in effect resulting in an underestimation of the chances to achieve a favorable outcome. In addition, reporting the number of patients treated with cranioplasty would have been of additional value to the study results and constitutes another drawback. However, as cranial reconstruction is a known important factor for functional recovery, the expected effect of outcome assessments possibly based on a few patients still harboring a cranial defect would be an underestimation of the overall results and chances of a favorable outcome after DC in aSAH. Thus, an outcome assessment beyond six-months post-ictus (probably at least twelve months) would for several reasons have been more appropriate to capture the actual long-term outcome in these patients. Moreover, as part of a broader study project on DCs, the study did not include a reference group of aSAH patients managed conservatively. This is important to bear in mind when interpreting the results, as some patients in poor condition (or for other various reasons) were probably disqualified for DC and, had they been treated, could theoretically have attenuated the somewhat favorable results demonstrated. While the analyses utilized detailed clinical and radiological data routinely employed in clinical practice, only age emerged as a significant predictor of six-months outcomes. A more nuanced radiological assessment of the extent and neuroanatomical location of each brain injury could have added further predictive information and should likely be considered in future research attempts aiming to understand outcomes after DC for aSAH. Although this study constitutes one of the largest cohorts to date on aSAH patients treated with DC, the treatment remains rare. Consequently, the statistical power of the analyses – particularly regarding mortality and favorable outcome predictors – was limited. To enable more robust analyses and develop firmer multivariable regression models, larger cohorts are required.

## Conclusions

DC in patients with aSAH demonstrated immediate radiological reduction of mass effect and a low incidence of complications requiring reoperation. Despite the severe underlying brain injuries predisposing patients to intracranial hypertension, 80% survived at six months, with 33% achieving favorable outcome. Younger age was identified as the strongest predictor of favorable outcomes, while other patient- and injury-specific factors offered limited prognostic value. These results underscore the potential for meaningful recovery in a subset of aSAH patients treated with DC. However, the absence of robust predictors beyond age complicates the selection of candidates for DC in cases of refractory intracranial hypertension. Future prospective studies are needed to refine the indications and patient selection for DC and to identify factors associated with optimal outcomes.

## Data Availability

Data are available upon reasonable request.
